# Fidelity and the impact of patient safety huddles on teamwork and safety culture: an evaluation of the Huddle Up for Safer Healthcare (HUSH) project

**DOI:** 10.1186/s12913-021-07080-1

**Published:** 2021-10-01

**Authors:** Laura Lamming, Jane Montague, Kate Crosswaite, Muhammad Faisal, Eileen McDonach, Mohammed A. Mohammed, Alison Cracknell, Alison Lovatt, Beverley Slater

**Affiliations:** 1 Faculty of Health Studies, University of Bradford, Bradford, UK; 2 Salford Royal NHS Foundation Trust Manchester, England, UK; 3 St James’s University Hospital, Leeds Teaching Hospitals Trust, Leeds, UK; 4 The Improvement Academy, Bradford Institute for Health Research, Bradford, UK

**Keywords:** Patient safety, Patient safety huddles, Teamwork and safety culture, Fidelity

## Abstract

**Background:**

The Patient Safety Huddle (PSH) is a brief multidisciplinary daily meeting held to discuss threats to patient safety and actions to mitigate risk. Despite growing interest and application of huddles as a mechanism for improving safety, evidence of their impact remains limited. There is also variation in how huddles are conceived and implemented with insufficient focus on their fidelity (the extent to which delivered as planned) and potential ways in which they might influence outcomes. The Huddle Up for Safer Healthcare (HUSH) project attempted to scale up the implementation of patient safety huddles (PSHs) in five hospitals – 92 wards - across three UK NHS Trusts. This paper aims to assess their fidelity, time to embed, and impact on teamwork and safety culture.

**Methods:**

A multi-method Developmental Evaluation was conducted. The Stages of Implementation Checklist (SIC) was used to determine time taken to embed PSHs. Observations were used to check embedded status and fidelity of PSH. A Teamwork and Safety Climate survey (TSC) was administered at two time-points: pre- and post-embedding. Changes in TSC scores were calculated for Trusts, job role and clinical speciality.

**Results:**

Observations confirmed PSHs were embedded in 64 wards. Mean fidelity score was 4.9/9. PSHs frequently demonstrated a ‘fear free’ space while Statistical Process Control charts and historical harms were routinely omitted. Analysis showed a positive change for the majority (26/27) of TSC questions and the overall safety grade of the ward.

**Conclusions:**

PSHs are feasible and effective for improving teamwork and safety culture, especially for nurses. PSH fidelity criteria may need adjusting to include factors deemed most useful by frontline staff. Future work should examine inter-disciplinary and role-based differences in TSC outcomes.

**Supplementary Information:**

The online version contains supplementary material available at 10.1186/s12913-021-07080-1.

## Background

Avoidable patient safety harm remains a problem within healthcare in the United Kingdom (UK). About 8% of patients in the National Health System (NHS) have experienced an adverse event [1]. Between October 2017 and September 2018, for example, English NHS organisations reported 1,991,797 incidents as occurring. This is 5.1% more than between October 2016 and September 2017 (1,895,834).

Recent attempts to reduce avoidable harms in hospitals include Patient Safety Huddles (PSHs): brief, daily, multidisciplinary meetings that allow teams to convene, review and ensure safe care [2]. Goldenhar et al. have defined huddles as: “ … typically short briefings designed to give frontline staff and bedside caregivers opportunities to stay informed, review events, make and share plans for ensuring well-co-ordinated patient care.” [2] They draw on the practice of High Reliability Organisations (HROs) such as in the nuclear and aviation industries [3]. PSHs are reported as versatile, relatively low cost interventions which have a positive impact on patient safety [4–7]. Originating in North America, but increasingly implemented in the UK healthcare settings PSHs can address threats to patient safety, such as those measured by the NHS Safety Thermometer: pressure ulcers, falls, infection from urinary catheters and venous thrombus embolism.^1^ PSHs aim, and have been shown to, improve safety in part by improving staff communication and teamwork which contribute to safety climate [8–11].

In February 2015, following a scaling up award from The Health Foundation (THF), a major project to scale up PSHs named HUSH (Huddle Up for Safer Healthcare)^1^ began in five hospitals (three National Health Service (NHS) Trusts) in the Yorkshire & Humberside region of England. The huddles were conceived as a complex intervention, specifically for hospital-based teams and sought to enhance team-working and safety climate in the ward environment and thereby reduce patient harms (e.g. falls).

The HUSH team described their particular PSH as “… a ‘vehicle’ for daily, brief, frontline, non- hierarchical, multi-disciplinary, focussed discussion of a specific patient harm, led by senior clinical management and supported by quality improvement skills, coaching, data visualisation and feedback.” (Updated Evaluation Protocol, Scaling Up Safety Huddles, February 2015 [12]). This initial description was based on preliminary insights from eight pilot wards.

Coproduced by the HUSH implementation team, the operational definition of a PSH is as follows:

1. Takes place at the same venue and time every day
2. Is led by the most senior clinician
3. Includes a review of the number of days since the last harm
4. Includes a review of an improvement run chart
5. Includes a de-brief of any harms since the last huddle
6. Includes discussion of who is at risk today and what needs to be put in place
7. Participants are asked if anyone has any other concerns
8. Is short and sweet (≤0–15 min)
9. Is a non-judgemental and fear-free space.

Subsequently, the HUSH project team implemented PSHs across three NHS Trusts, in adult in-patient wards in five acute hospitals. This paper reports on the impact of PSHs on teamwork and safety culture, along with fidelity (which is under-researched [6]). Dumas et al. [13] define fidelity as ‘ the demonstration that an experimental manipulation is conducted as planned’. Fidelity is deemed to have been achieved if each element of the intervention is delivered without variation. This enables the intervention to be repeated, evaluated, compared and disseminated and is therefore crucial for any further implementation [14]. The team’s operational definition of a PSH (above) were used as fidelity criteria in this instance.

## Methods

### Sample

The HUSH project followed on from a successful huddle implementation pilot of eight wards at Leeds NHS Trust. The HUSH implementation team set out to scale up PSHs in 136 inpatient wards in three Yorkshire and Humberside NHS Trusts. This number was eventually revised because of ward closures, mergers and exclusions and so 92 wards were included in the evaluation (see Additional file 1). Of these 92 wards, 66 PSHs were observed in 64 wards. Fidelity data relates to 66 observations on 64 wards. (On two of the wards, two PSHs were observed consecutively: on one ward the PSH observation took place in different bays and for the other ward, two huddles took place at different times). The primary unit of analysis is an observed PSH (*n* = 66). TSC survey data relates to those wards that completed the survey.

### Evaluation

A multi-method Developmental Evaluation [15, 16] over a three year period was undertaken to assess the implementation, fidelity, effectiveness, return on investment and learning from scaling up of PSHs. This included data collection from multiple sources and ‘Evaluation Dress Rehearsals’. The latter facilitated feedback on data analysis, double-loop learning – learning that both informs the implementation and its evaluation - and discussion of issues as they emerged within implementation teams. Methods are reported for assessment of fidelity and teamwork and safety climate.

### Implementation and fidelity

The Stages of Implementation Checklist (SIC), adapted from the Stages of Implementation Completion tool [17] was used to determine length and timing of phases of implementation and how long it took for a ward to embed PSHs (Additional file 2). Structured observations confirmed embedded status – wards that had held 15 PSHs in < 21 days - based on SIC and fidelity based on the nine characteristics coproduced by the HUSH implementation team and informed by early insights from pilot wards (Table 1). These criteria were based on face validity, but subject to revision over time, unsurprisingly, given the complexity of developing the intervention. Fidelity data relates to observations of 66 PSH on 64 wards and our TSC survey data relates to those wards that took part that completed the survey.

**Table 1:** Frequency of nine PSH characteristics observed on embedded wards

Fidelity criteria for PSHs	Number of PSHs observed with each criteria (66 observations on 64 wards)
1. Same place and time	53 (80%)
2. Led by most senior clinician	6 (9%)
3. Review of number of days since last harm conducted	18 (28%)
4. Review of improvement run chart conducted	0 (0%)
5. Debrief of any harm since last huddle	23 (35%)
6. Discussion of who is at risk today and what needs to be put in place	63 (95%)
7. Participants asked if anyone has any other concerns about patients	37 (56%)
8. Is PSH short and sweet	61 (92%)
9. Non-judgemental ‘fear-free’ space	64 (97%)

### Teamwork and safely climate

A validated Teamwork and Safety Climate survey (TSC) was administered to ward staff by members of the HUSH implementation team at two time-points: during the pre-embedded phase (before implementation if feasible) and post-embedded. Additional file 3 shows the number of responders from each ward pre and post. We did not record the number of forms that were distributed and so are unable to determine response rates.

Based on the Safety Attitudes Questionnaire, it consisted of 27 Likert-scale questions from disagree strongly- to agree strongly [18]. An additional question from the Agency for Healthcare Research and Quality Hospital Survey on Patient Safety Culture (SOPS™) [19] requested an overall assessment of patient safety on the ward (Additional file 4). As the four questions (Q2, Q6, Q8, Q23) were inversed in the TSC survey, we reversed them for the analysis and interpretation purpose. Ward staff completed the survey anonymously, but provided details of their job role. It was not possible to ensure the same staff participated at both time points.

### Analysis

Change in TSC scores between pre and post embeddedness was calculated using the Generalised Estimating Equation, with robust standardised errors and the ward as the clustering variable. Likert scales were recoded to a continuous variable (1-low, 5-high) and a binary covariate was used in the statistical model (0 = pre embedded versus 1 = post embedded). An exchangeable correlation structure was assumed. Analysis was by Trust, speciality (medicine, surgery, critical care and other) and job role (nurse, doctor, allied health professional, nursing support staff, ward support such as clerk or housekeeper, and other). Ninety-five percent confidence intervals are reported.

### Ethics and approvals

Ethical approval was received from the Chair of the University of Bradford Biomedical, Natural, Physical and Health Sciences Research Ethics Panel in March 2016 (EC2230). The Research and Development Departments at each Trust confirmed it was an ‘evaluation or service review’ by March 2016, and did not require NHS ethical review. Honorary contracts and written permissions were obtained to access wards. This project was funded by the first round of the Scaling Up call from The Health Foundation.

## Results

### Implementation and fidelity

Forty-four of 136 wards targeted for intervention were excluded (see Additional file 1). The remaining 92 wards were unequally distributed across the five hospitals, (range 3–38). According to the self-reported SIC, 75 (82%) had embedded PSHs. Across the five hospitals the percentage of wards where PSHs were embedded ranged from 78 to 100%. However, observations suggested PSHs were embedded in only 64 wards. Ward PSHs were considered embedded - following a period of implementation - when they had run 15 huddles in less than 21 days. The mean time for a ward to embed PSHs, based on the SIC, was 19.6 weeks – within the anticipated time of 24 weeks (range 1–86 weeks). Individually, hospitals ranged from a mean of 18 to 48 weeks to embed PSHs.

The mean fidelity score across 64 embedded wards was 4.9 (range = 3–8). No ward reviewed their run charts. The most frequently observed criterion was a non-judgemental ‘fear free’ space (64/66 observations of 64 wards), the least observed criterion was the PSH being led by the most senior clinician (6/66) (Table 1).

### Teamwork and safety climate survey

A total of 2850 responses to the TSC were captured, 1477 pre-embedded and 1373 post-embedded across 67/75 embedded wards (according to the SIC).

Mean percentage difference showed an overall positive trend for the majority of TSC survey questions between pre-embedded and post-embedded stages. The questions with the highest positive mean percentage difference are as follows: + 7.05% for Q19 (‘The culture in this clinical area makes it easy to learn from the errors of others’); + 6.85% for Q12 (‘Briefings are common in this clinical area’); + 5.82% for Q28 (‘Please give your unit an overall grade on patient safety’); and + 5.13% for Q8 (‘I have the support I need from other personnel to care for patients’) (see Table 2).

**Table 2:** Summary statistics of TSC survey questions at pre-embedded and post-embedded stage

Question	Pre-embedded		Post-embedded		Mean percentage difference
	Mean (SD)	Median (IQR)	Mean (SD)	Median (IQR)	
Q1 Nurse input is well received in this clinical area	4.31 (1.13)	5 (1)	4.43 (1.04)	5 (1)	▲ 2.68%
Q2 In this clinical area, it is difficult to speak up if I perceive a problem with patient care	3.84 (1.45)	4 (2)	4.01 (1.36)	5 (1)	▲ 4.39%
Q3 Decision-making in this clinical area utilises input from relevant personnel.	4.11 (1.22)	5 (1)	4.23 (1.15)	5 (1)	▲ 2.97%
Q4 The doctors and nurses here work together as a well-coordinated team	4.17 (1.16)	5 (1)	4.26 (1.12)	5 (1)	▲ 2.21%
Q5 Disagreements in this clinical area are resolved appropriately (i.e., not *who* is right, but *what* is best for the patient)	4.01 (1.27)	4 (2)	4.1 (1.24)	5 (1)	▲ 2.29%
Q6 I am frequently unable to express disagreement with the medical staff here	3.5 (1.5)	4 (2)	3.63 (1.5)	4 (2)	▲ 3.83%
Q7 It is easy for personnel here to ask questions when there is something that they do not understand.	4.37 (1.08)	5 (1)	4.45 (1.04)	5 (1)	▲ 1.88%
Q8 I have the support I need from other personnel to care for patients	4.12 (1.3)	5 (1)	4.33 (1.18)	5 (1)	▲ 5.13%
Q9 I know the first and last names of all the personnel I worked with during my last shift	3.69 (1.51)	4 (3)	3.77 (1.45)	4 (2)	▲ 2.14%
Q10 Important issues are well communicated at shift changes’	3.93 (1.45)	4 (1)	4.14 (1.31)	5 (1)	▲ 5.39%
Q11 Briefing personnel before the start of a shift (i.e. to plan for possible contingencies) is important for patient safety	4.42 (1.25)	5 (1)	4.56 (1.12)	5 (0)	▲ 3.04%
Q12 Briefings are common in this clinical area	4.1 (1.28)	5 (1)	4.38 (1.11)	5 (1)	▲ 6.85%
Q13 I am satisfied with the quality of collaboration that I experience with medical staff in this clinical area	4.03 (1.19)	4 (1)	4.13 (1.2)	5 (1)	▲ 2.59%
Q14 I am satisfied with the quality of collaboration that I experience with nurses in this clinical area	4.28 (1.09)	5 (1)	4.4 (1.06)	5 (1)	▲ 2.86%
Q15 The levels of staffing in this clinical area are sufficient to handle the number of patients	2.57 (1.45)	2 (3)	2.69 (1.45)	2 (3)	▲ 4.82%
Q16 I would feel safe being treated here as a patient	3.8 (1.3)	4 (2)	3.93 (1.26)	4 (2)	▲ 3.56%
Q17 I am encouraged by my colleagues to report any patient’s safety concerns I might have	4.35 (1.1)	5 (1)	4.47 (0.99)	5 (1)	▲ 2.77%
Q18 Personnel frequently disregard rules or guidelines (e.g. hand washing, treatment protocols/clinical pathways, sterile fluid, etc.) that are established for this clinical area.	3.7 (1.54)	4 (2)	3.74 (1.5)	4 (2)	▲ 0.97%
Q19 The culture in this clinical area makes it easy to learn from the errors of others	3.67 (1.33)	4 (2)	3.92 (1.24)	4 (2)	▲ 7.05%
Q20 I received appropriate feedback about my performance	3.71 (1.39)	4 (2)	3.82 (1.35)	4 (2)	▲ 3.18%
Q21 Medical errors are handled appropriately here.	3.92 (1.41)	4 (2)	3.98 (1.43)	5 (2)	▲ 1.52%
Q22 I know the proper channels to direct questions regarding patient safety in this clinical area	4.32 (1.02)	5 (1)	4.43 (0.95)	5 (1)	▲ 2.48%
Q23 In this clinical area, it’s difficult to discuss errors	3.72 (1.34)	4 (2)	3.84 (1.32)	4 (2)	▲ 3.12%
Q24 Hospital management does not knowingly compromise the safety of patients	3.22 (1.48)	3 (3)	3.21 (1.49)	3 (3)	▼ 0.21%
Q25 This organisation is doing more for patient safety now, than it did one year ago	3.37 (1.59)	4 (2)	3.49 (1.54)	4 (2)	▲ 3.67%
Q26 Leadership is driving us to be a safety-centred organisation	3.79 (1.23)	4 (2)	3.86 (1.22)	4 (2)	▲ 1.82%
Q27 My suggestions about safety would be acted upon if I expressed them to management	3.69 (1.28)	4 (2)	3.77 (1.29)	4 (2)	▲ 2.26%
Q28 Please give your unit an overall grade on patient safety	3.81 (0.89)	4 (1)	4.03 (0.84)	4 (1)	▲ 5.82%

*Note: SD* Standard deviation; *IQR* Interquartile range

**Fig. 1:**
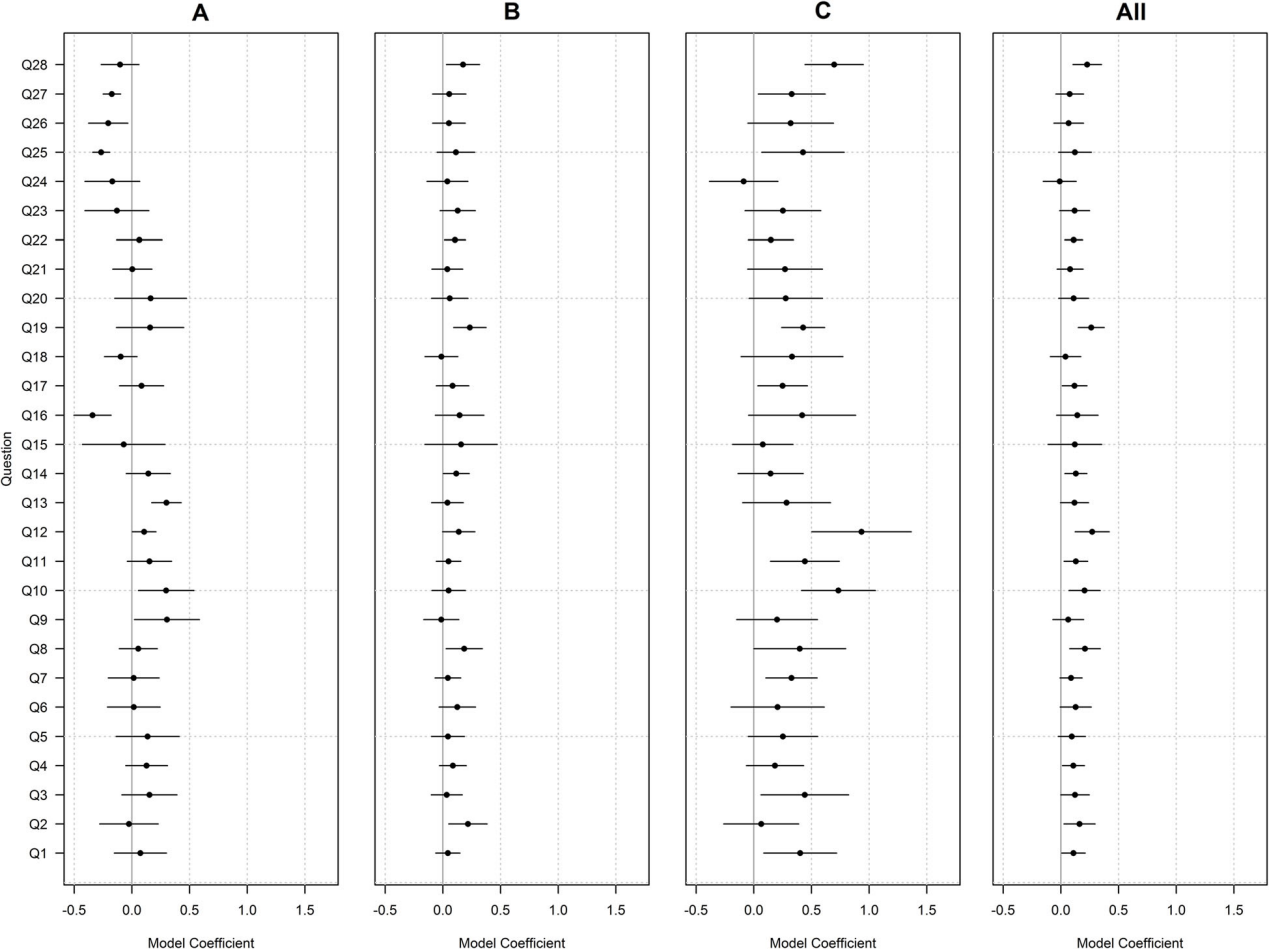
TSC results per question by Trust: Model coefficients above zero show a positive change, below zero show a negative change. Horizontal lines indicate 95% CI

Statistical modelling results also showed a general positive change for the majority of questions between time points for each Trust, with questions Q8 (‘I have the support I need from other personnel to care for patients’); Q12 (‘Briefings are common in this clinical area’); Q19 (‘The culture in this clinical area makes it easy to learn from the errors of others’) and Q28 (‘Please give your unit an overall grade on patient safety’) showing the largest change (Fig. 1). Trusts B and C showed predominantly positive changes, while Trust A showed mixed changes. The degree of improved responses varied between questions, however Trusts reported some of their largest improvements for Q28 and Q19 (B and C) and Q12 (C). Results by hospital are not reported due to small sample sizes.

**Fig. 2:**
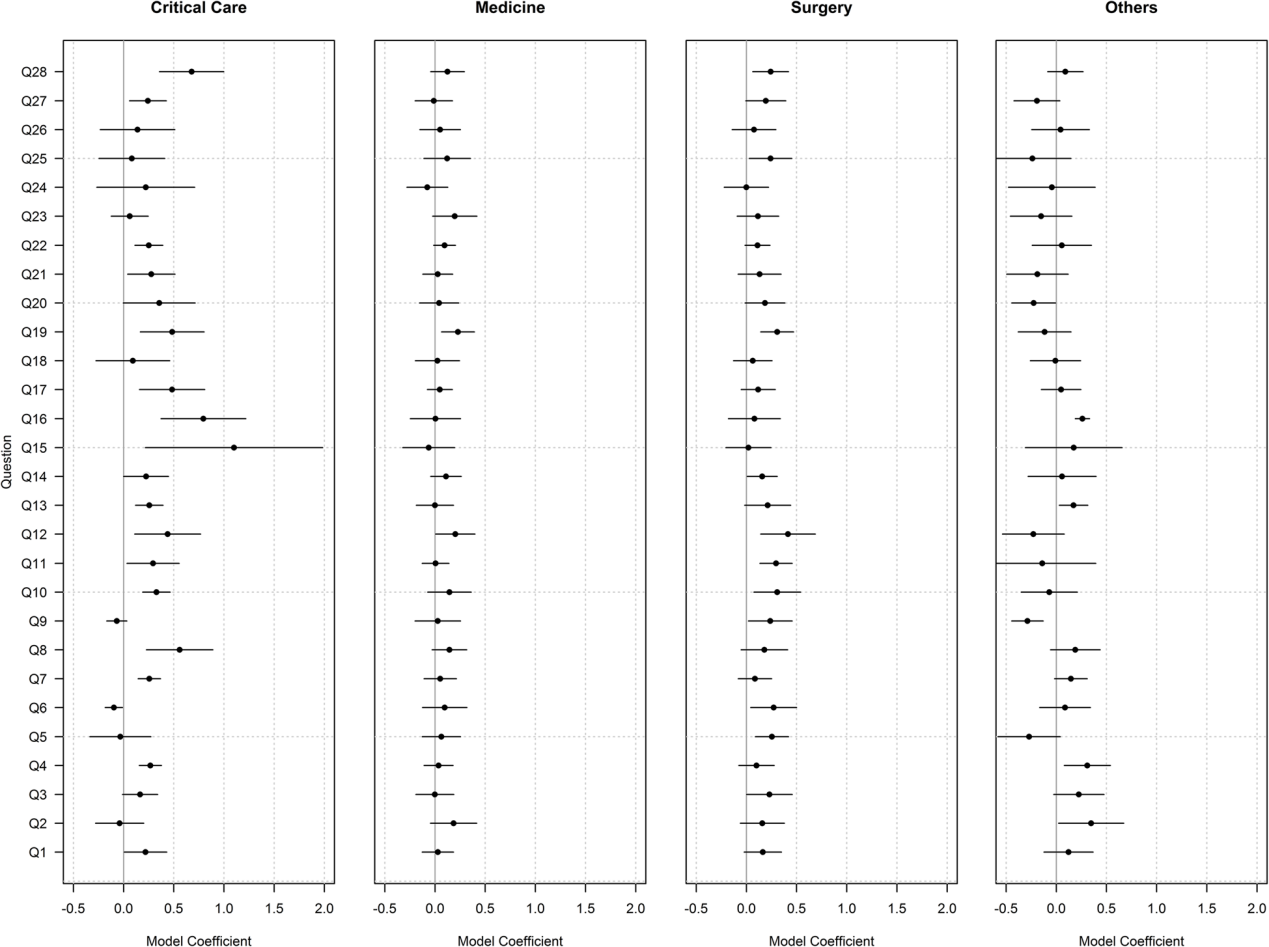
TSC results by Speciality: Model coefficients above zero show a positive change, below zero show a negative change. Horizontal lines indicate 95%C

Staff on critical care wards (*n* = 327, 11.5%) showed the greatest positive changes across the most questions with Q15 (‘The levels of staffing in this clinical area are sufficient to handle the number of patients’) showing the largest positive change and only Q6 (‘I am frequently unable to express disagreement with the medical staff here) showing a negative change. Staff on surgical wards (n= 1051, 36.9%) showed a positive change across nine questions, the largest of which was in Q12 and no negative changes. Staff on medical wards (n=1226, 43%) showed positive change in Q12 and Q19. Staff from ‘other’ specialities (n=246, 8.6%) showed a negative change in Q9 (‘I know the first and last names of all the personnel I worked with during my last shift’) and a positive change in Q16 (‘I would feel safe being treated here as a patient’). Both critical care and surgical wards reported improvements in Q28 (Fig. 2).

**Fig. 3:**
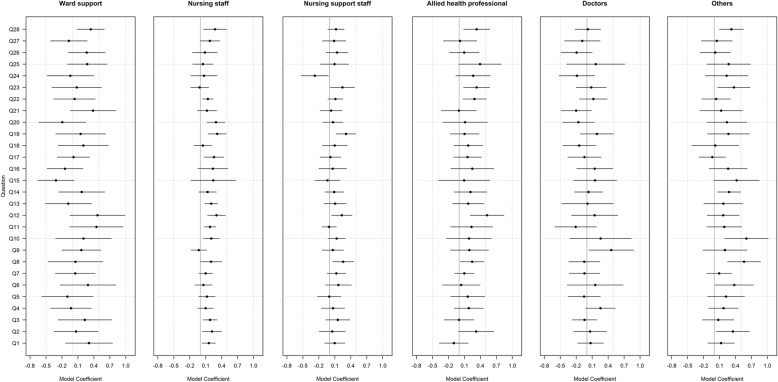
TSC results by Job Role: Model coefficients above zero show a positive change, below zero show a negative change. Horizontal lines indicate 95% CI

All staff except doctors (*n* = 202, 7.3%) showed a positive shift in their overall assessment of the safety of the unit (Q28). Nurses (*n* = 1149, 41.7%) showed ten positive changes, the largest for Q12, Q17 (‘I am encouraged by my colleagues to report any patient’s safety concerns I might have’), Q19 and Q20 (‘I received appropriate feedback about my performance’). Doctors showed the largest positive changes for Q9. Allied Health Professionals (*n* = 248, 9%) showed the largest positive change in Q12, Q22 (‘I know the proper channels to direct questions regarding patient safety in this clinical area’) and Q23 ‘In this clinical area, it’s difficult to discuss errors’). Nursing support staff (*n* = 655, 23.7%) saw positive changes in Q8, Q12, Q19 and Q23, and were the only group to report a negative change, in Q24 (‘Hospital management does not knowingly compromise the safety of patients’). Ward staff (*n* = 246, 8.9%) saw a positive change in Q28 only. Other staff (*n* = 259, 9.4%) saw a positive change in Q2 (‘In this clinical area, it is difficult to speak up if I perceive a problem with patient care’), Q8, Q10 (‘Important issues are well communicated at shift changes’), Q14 (‘I am satisfied with the quality of collaboration that I experience with nurses in this clinical area’), Q28 and Q23 (Fig. 3).

## Discussion

This paper has described the fidelity, time to embed and impact on teamwork and safety culture of patient safety huddles implemented in five hospitals across three UK NHS Trusts as part of The Huddle Up for Safer Healthcare project.

Consistent improvements occurred including: briefings being seen by staff as common, the culture making it easy to learn from others’ errors and the overall patient safety grade assigned to units by staff. These findings align with other studies showing that huddles improved both the number and quality of communication opportunities [2, 20]. However, that the improvement in overall patient safety grade was not reflected in improvements in other measurement items suggests the TSC may not reflect factors that staff consider when responding to this question.

Across 92 wards, the rates of embedded PSHs were high (64), taking an average 19.6 weeks to embed. No ward demonstrated all fidelity criteria as originally described by the HUSH implementation team; an average of 4.9/9 criterion was observed. A non-judgemental ‘fear free’ space was observed in almost all PSHs but run chart completion was never observed. Teamwork and Safety Culture scores tended to improve over time across all Trusts. The greatest improvements across the most items were seen in critical care wards but reductions in some items were also seen. The majority of staff reported positive changes but this varied by job role and by TSC item. Nurses in particular demonstrated the most positive changes while doctors showed very few: they were the only staff group who did not relate improved ratings of the safety of their unit. Overall, the findings suggest that embedding PSHs is feasible and effective for changing TSC scores but fidelity to the full set of originally designated criteria is moderate. Therefore, certain criteria may be less essential for promoting a positive teamwork and safety climate and could therefore be adapted as necessary.

The current findings support studies showing that implementing huddles is feasible, effective, and huddles themselves are acceptable to hospital staff [9, 11, 20]. Given the extensive barriers to successfully scaling up a quality improvement initiative - for example, the politics of organisations, user engagement and the role of the team [21]- the number of wards that achieved embedded status can be considered a success. However, that moderate PSH fidelity was observed suggests some of the original criteria were problematic for staff. Quality improvement literature emphasises the importance of programme fidelity as the degree to which the initiative is implemented is a potential moderator of its effect [22]. Failing to have a PSH led by a senior clinician may appear to be of concern (only 9% of wards succeeded) but, given the high rate of embedding, it does not appear to have worked against the initiative. In fact, this may have facilitated the frequency with which a ‘non-judgemental ‘fear free’ space’ was observed. Therefore, while an effective leader is key for fidelity as in a high performing hospital, [23, 24], the level of seniority may not be.

Of more concern was the poor uptake of the review of the number of days since the last harm (28%), debrief of any harm since the last huddle (35%) and the review of run charts (0%), all of which involve an assessment of the ward’s recent history of safety/harms. The HUSH implementation team speculated that some criteria may be more or less central to a successful PSH and that PSHs would be adapted to the ward team’s needs. It may be the case that staff are reluctant to focus on past harms for fear of reprisal. Cohen et al. (2003) surveying nurse attitudes to medication error reporting found that staff were fearful of reporting errors and subsequently being perceived as a poorer nurse and/or having a blemish on their record. More generally, Okuyama et al. (2014) [25] found that staff are reluctant to voice safety concerns for multiple reasons, including discipline, efficacy and responses of others. However, the frequency with which a ‘non-judgemental ‘fear free’ space’ was observed refutes the proposal that staff were fearful of reprisal. Criteria that were consistently observed may have been simpler to achieve, pragmatic, and perceived as more relevant. Further research should explore these hypotheses and determine whether certain criteria are deemed more or less useful by frontline staff.

Nurses perceived more improvements in TSC items than other staff groups, especially doctors. It is possible that while nurses saw the benefits of regular, current, short and fear free forums to their own practice and therefore the culture of their unit, doctors required different PSH criteria to experience culture change. For example, reviewing historical harms, run charts and days since last harm may have been more demonstrative of culture change for this group, as they would provide a concrete demonstration of improvements in ward safety. Alternatively, the ward-based nature of nurses, compared to doctors who tend to move between wards, might mean that nurses are better placed to observe subtle improvements in communication and culture. Importantly, the poor PSH fidelity may explain why changes in TSC, though positive, were not consistently so across wards and staff roles.

Some ward characteristics identified by the TSC still need improvement. HUSH shows infrequent improvements in some of these factors but these could be built on: for example, staffing levels, reporting concerns/events and difficulty in discussing errors. Reis et al. (2018) [26] in a worldwide study of the need for safety culture improvement suggest that the latter could be linked to a culture of blame. In addition, the few negative changes in some TSC items may reflect raised awareness of these factors. Aldawood et al. (2020) [20] found that the use of patient safety huddles served to increase awareness of and improvements in safety culture. Critical care wards indicated an increase in frequently being unable to express disagreement; given the high-stakes nature of critical care wards, huddles may have increased perception of a necessarily highly hierarchical setting. Other wards reported an increase in not knowing staff names, which PSHs may have made more apparent. Nursing support staff reported a reduction in the perception that the hospital did not knowingly compromise safety. It may be that by taking part in regular huddles with a range of senior staff who are more familiar with addressing them, they may have become more aware of safety issues, how they occur and how they are managed [20].

### Limitations

The HUSH study had limitations. HUSH was not the only quality improvement initiative running in the hospitals and therefore it is possible that outcomes were confounded by other initiatives. However, other studies have found that huddles are typically a component of a wider patient safety programme, so this is not unusual [27]. In addition, Kristensen et al. (2015) [28] suggest that there are positive associations between the implementation of quality management systems and improvements in teamwork and safety culture.

A second limitation was that, at the outset, the nine characteristics of a PSH defined in this study were deemed to be of equal weighting by the team which is not an unreasonable preliminary notion given the acknowledged advantages of the use of equal weights [29]. The average scores were based on the initial assumption that each criterion was equally important for staff. However, as we learned about how PSHs were being implemented and adapted over time we could see the need to review, revise and also rank these criteria by importance (see Table 3). In addition, a new PSH characteristic was included: ‘a range of staff including non-clinical’ (30/01/2018). (see [30]).

**Table 3:** Revised criteria for PSH operational definition (source: HUSH Team)

Most important:	
• Who is at risk today and what needs to be put in place	
• Non-judgemental (staff feel free to speak up)	
• A range of staff including non-clinical	
Important:	
• Same place and time	
• Review of days since last harm	
• Review of harm events since last huddle (team de-brief)	
• Huddle is short and sweet	
• Any other concerns about patients	
Moderate importance:	
• Led by a credible Healthcare professional	

Another potential limitation was the use of Developmental Evaluation: having the evaluation team working closely with, and providing feedback to, the implementation team. This meant that the evaluation should be considered as part of the intervention itself, meaning outcomes may have been confounded by the evaluation process. In turn, the evaluation was impacted by the implementation – with the order of ward recruitment continually fluctuating due to coach availability, staffing changes in both the HUSH team and ward staff, and other practical issues such as ward mergers.

Additionally, there was both a lack of engagement as well as early enthusiasm from some wards. While the evaluation team responded flexibly, some planned data collection opportunities had to change and some wards were lost over time. For example, the first TSC was delivered after implementation began, but before the huddles were embedded. A final limitation was that we were not able to determine the response rate for the TSC survey, although anecdotally, the team felt that in some wards it was low.

### Implications for practice

Overall, the findings suggest that PSHs are feasible and effective for improving teamwork and safety culture in a busy, ever-changing hospital context, particularly for nursing staff. Huddles do not lead to an overall increase in ward workload and the cost of supporting the huddle are small compared to the savings per harm (see Crosswaite et al. 2018 [31] for a ROI analysis). However, all nine huddle criteria, as originally described by the implementation team, may not be essential to achieve the described positive changes. These findings have three possible implications, one is that huddles could be adapted to include only the most relevant criteria as deemed by frontline staff. Such changes could capitalise on the observed improvements in TSC, producing a larger change both within and across TSC items. Secondly, the TSC may not be appropriate for all clinical areas. The fact that critical care wards felt that they were frequently unable to express disagreement may be a symptom of the organisational structure rather than a genuine barrier for patient safety. Thirdly, PSHs may be particularly appealing and beneficial for nurses as it gives them a regular multi-disciplinary forum for shared communication, reporting and feedback. However, while change in TSC may be facilitated by PSHs, huddles that neglect certain criteria may be insufficient for doctors to recognise the changes in TSC; TSC may raise awareness of poor ward characteristics and therefore further or longer intervention of PSHs may be required. Future work should explore whether the TSC should be specialised for different clinical areas and staff roles or if there is scope for change in communication practices.

## Conclusions

Patient safety huddles are a feasible intervention to improve teamwork and safety culture in hospitals, especially among nurses. The most consistent changes were seen in perceptions that briefings were common, the culture made it easy to learn from others’ errors, and the overall safety grade of the ward. However, the latter was not reflected in changes across other measurement items, questioning the factors that influence this decision. The defining criteria of PSH may need changing to those deemed most useful by staff – an important influence on outcomes - for different staff groups. We believe that this a major point of our paper, that fidelity criteria may change subject to revision based on experience and evidence. Acknowledging this as a possibility at the outset is important. Future work should determine if TSC items raise awareness of poor ward cultures among wards implementing PSHs, as well as measuring improvements in safety culture.

## Supplementary Information

**Additional file 1.** Reasons for ward exclusions.

**Additional file 2.** Stages of Implementation checklist.

**Additional file 3.** Sample of TSC survey sizes: pre and post embedded.

**Additional file 4.** Teamwork and Safety Culture survey.

## Data Availability

The datasets used and/or analysed during the current study are available from the corresponding author on reasonable request.

## Footnotes

1 www.safetythermometer.nhs.uk/index.php/classic-thermometer

## References

[1] Improvement NHS (2019). NRLS National Patient Safety Incident Reports: commentary [internet].

[2] Goldenhar LM, Brady PW, Sutcliffe KM, Muething SE (2013). Huddling for high reliability and situation awareness. BMJ Qual Saf.

[3] Weick KE, Sutcliffe KM, Obstfeld D, Sutton RS, Staw BM (1999). Organising for high reliability: processes of collective mindfulness. Research in organizational behavior.

[4] Sikka R, Kovich K, Sacks L (2014). How Every Hospital Should Start the Day [Internet]. Harvard Business Review.

[5] Larsen D, Peters H, Keast J, Devon R (2011). Using real time patient feedback to introduce safety changes. Nurs Manag.

[6] Franklin BJ, Gandhi TK, Bates DW, Huancahuari N, Morris CA, Pearson M (2020). Impact of multidisciplinary team huddles on patient safety: a systematic review and proposed taxonomy. BMJ Qual Saf.

[7] Venkataraman A, Conn R, Cotton RL, Abraham S, Banaghan M, Callaghan B (2018). Perspectives of multidisciplinary staff toward the Improvement of communication and patient safety by safety huddles. Patient Saf Qual Improv J.

[8] Glymph DC, Olenick M, Barbera S, Brown EL, Prestianni L, Miller C (2015). Healthcare utilizing deliberate discussion HUDDLE. AANA J [Internet].

[9] Wilbur K, Scarborough K (2005). Medication safety huddles: teaming up to improve patient safety. Can J Hosp Pharm.

[10] Gerke ML, Uffelman C, Weber CK. Safety huddles for a culture of safety. Patient Saf Qual Healthc. 2010.

[11] Donnelly LF, Cherian SS, Chua KB, Thankachan S, Millecker LA, Koroll AG, Bisset GS (2017). The daily readiness huddle: a process to rapidly identify issues and foster improvement through problem-solving accountability. Pediatr Radiol.

[12] McDonach E, Crosswaite K, Mohammed M. Mixed methods, developmental evaluation of a quality improvement initiative to scale up a complex intervention, patient safety huddles (PSH), across three acute hospital trusts in Yorkshire: updated evaluation protocol. Bradford: University of Bradford; 2015.

[13] Dumas JE, Lynch AM, Laughlin JE, Smith EP, Prinz RJ (2001). Promoting intervention fidelity conceptual issues, methods, and preliminary results from the EARLY ALLIANCE prevention trial. Am J Prev Med.

[14] Musuuza JS, Barker A, Ngam C, Vellardita L, Safdar N (2016). Assessment of fidelity in interventions to improve hand hygiene of healthcare workers: a systematic review. Infect Control Hosp Epidemiol.

[15] Patton MQ (2011). Developmental evaluation. Applying complexity concepts to enhance innovation and use.

[16] Patton MQ (2006). Evaluation for the way we work. Nonprofit Q.

[17] Saldana L (2014). The stages of implementation completion for evidence-based practice: protocol for a mixed methods study. Implement Sci.

[18] Sexton JB, Helmreich RL, Neilands TB, Rowan K, Vella K, Boyden J (2006). The Safety Attitudes Questionnaire: psychometric properties, benchmarking data, and emerging research. BMC Health Serv Res.

[19] Sorra J, Gray L, Streagle S, Famolaro T, Yount N, Behm J. AHRQ Hospital Survey on Patient Safety Culture: User’s Guide. AHRQ Publi. Westat, editor. Rockville, MD: Agency for Healthcare Research and Quality; 2018. 1–50 p.

[20] Aldawood F, Kazzaz Y, AlShehri A, Alali H, Al-Surimi K (2020). Enhancing teamwork communication and patient safety responsiveness in a paediatric intensive care unit using the daily safety huddle tool. BMJ open Qual.

[21] Allbury D, Beresford T, Dew S, Horton T, Illingworth J, Langford K (2018). Against the odds: Successfully scaling innovation in the NHS. Heal Found [Internet].

[22] Carroll C, Patterson M, Wood S, Booth A, Rick J, Balain S. A conceptual framework for implementation fidelity. Implement Sci 2007 [cited 2017 Nov 15];2(1):40. Available from: http://implementationscience.biomedcentral.com/articles/10.1186/1748-5908-2-40 10.1186/1748-5908-2-40 PMC2213686 18053122

[23] Taylor N, Clay-Williams R, Hogden E, Braithwaite J, Groene O (2015). High performing hospitals: A qualitative systematic review of associated factors and practical strategies for improvement. BMC Health Serv Res.

[24] Zaheer S, Ginsburg LR, Wong HJ, Thomson K, Bain L (2018). Importance of safety climate, teamwork climate and demographics: understanding nurses, allied health professionals and clerical staff perceptions of patient safety. BMJ Open Qual.

[25] Okuyama A, Wagner C, Bijnen B (2014). Speaking up for patient safety by hospital-based health care professionals: a literature review. BMC Health Serv Res.

[26] Reis CT, Paiva SG, Sousa P. The patient safety culture: a systematic review by characteristics of Hospital Survey on Patient Safety Culture dimensions. Int J Qual Health Care. 2018;30(9):660-77. https://academic.oup.com/intqhc/advance-article/doi/10.1093/intqhc/mzy080/4998840 10.1093/intqhc/mzy080 29788273

[27] Stockmeier C, Clapper C. Daily Check-In for Safety : From Best Practice to Common Practice. Patient Saf Qual Health; 2011.

[28] Kristensen S, Hammer A, Bartels P, Suñol R, Groene O, Thompson CA, Arah OA, Kutaj-Wasikowska H, Michel P, Wagner C (2015). Quality management and perceptions of teamwork and safety climate in European hospitals. Int J Qual Heal Care.

[29] Dawes RM (1979). The robust beauty of improper linear models in decision making. Am Psychol.

[30] Crosswaite K, Montague J, Mohammed M, Crosswaite K, Faisal M, Craig J, Marsh C, McDonach E, Mohammed MA (2018). An Evaluation of Eight Wards that Pioneered Safety Huddles. Huddling Up for Safer Healthcare (HUSH) The Health Foundation.

[31] Crosswaite K, Faisal M, Craig J, Marsh C, McDonach E, Mohammed MA (2018). Final report of the evaluation of the huddling up for safer healthcare (HUSH) scaling up project.

