# Supplementary material for: Fidelity and the impact of patient safety huddles on teamwork and safety culture: an evaluation of the Huddle Up for Safer Healthcare (HUSH) project
Source: BMC Health Serv Res. 2021 Oct 1;21:1038. doi: 10.1186/s12913-021-07080-1 (PMC8487146; doi:10.1186/s12913-021-07080-1)
Supplement: Supplementary file 1 — Additional file 1. Reasons for ward exclusions. [file 12913_2021_7080_MOESM1_ESM.docx]

**Appendix 1: Ward Exclusions**

What proportions of wards embedded PSHs?

There were 92 wards in the evaluation. Of these, four wards (4%=4/92) did not commence PSHs. Seventy five wards (82%=75/92) were noted as being embedded on the operational plan, but on independent observations 64 wards (85%=64/75, 70%=64/92) were found to be undertaking a PSH. Fifteen percent (=11/75) of the embedded wards were found to not have a PSH on the ward or not to be conducting a PSH on the day of the observation. Thirteen wards (14%=13/92) commenced PSHs but did not achieve embedded status of their PSH by the close of the evaluation (31.07.2017).

Four wards did not wish to huddle or faced major barriers at engagement. Barriers to, or a reluctance to implement huddles have been identified through general feedback and at operation meetings. The reasons for not implementing the PSH tended to fall into three main areas as follows:

- - 1. Ward leaders or teams stated that they did not wish to implement the PSH;
    2. Small units or wards (eight beds or less for example) that had a specialist remit and considered that they already have effective communication and multi-disciplinary working in place; and
    3. Ward leaders who considered that patient safety issues are adequately covered as part of the handover (at shift changes) or other ward based meetings.

The final number of wards in the evaluation was 92 (Table 1), of which 82% (75/92) were recorded by the HUSH team as having embedded PSHs over a two-year time period (starting: Aug 2015).

| **Ward set** | **Number of wards** |
| --- | --- |
| Number of wards in Operation Plan (31.07.2017) | **136** |
| Wards excluded | **44** |
| Wards included in this evaluation | **92** |
| Embedded wards | **75** |

**Table 1: Number of wards in the evaluation**

The table below shows the included/excluded wards by hospital. Scarborough General Hospital (SGH) had 100% (12/12) embedded PSHs, Leeds Trust (LTHT) including St James’s University Hospital (SJUH), Leeds General Infirmary (LGI) and Chapel Allerton Hospital (CAH) had 81% (52/64); and Barnsley General Hospital (BGH) (Barnsley NHS Foundation Trust) had 69% (11/16).

| **NHS Trust** | **Leeds** | **Leeds** | **Leeds** | **Barnsley** | **York** | **All** |
| --- | --- | --- | --- | --- | --- | --- |
| **Hospital** | **SJUH** | **LGI** | **CAH** | **BGH** | **SGH** |  |
| **Number of**  **wards** | N=50 | N=43 | N=3 | N=27 | N=13 | N=136 |
| **Wards excluded** | 12  Embedded cohort (n=8) Paediatric wards (n=1) Temporary wards (n=2) Day case ward (n=1) | 20  Paediatric wards (n=13) Day case wards (n=1) Ward moves (n=3)  Ward mergers  (n=3) | - | 11  Ward closure (n=1)  Paediatric wards (n=3) Ward move (n=1)  Ward mergers  (n=6) | 1  Paediatrics (n=1) | 44 |
| **Wards in the**  **evaluation** | 38 | 23 | 3 | 16 | 12 | 92 |
| **Embedded**  **wards** | 31 (81.5%) | 18 (78%) | 3 (100%) | 11 (69%) | 12 (100%) | 75 |

**Table 2: Number of: wards, excluded wards, evaluation wards and embedded evaluation wards (based on the Operation Plan 31/07/2017) by hospital**
