# Supplementary material for: Fidelity and the impact of patient safety huddles on teamwork and safety culture: an evaluation of the Huddle Up for Safer Healthcare (HUSH) project
Source: BMC Health Serv Res. 2021 Oct 1;21:1038. doi: 10.1186/s12913-021-07080-1 (PMC8487146; doi:10.1186/s12913-021-07080-1)
Supplement: Supplementary file 2 — Additional file 2. Stages of Implementation checklist. [file 12913_2021_7080_MOESM2_ESM.docx]

**Appendix 2: Stages of Implementation checklist**

The table below summarises the number of weeks from ‘start date of implementation to PSH embedded date’ for all embedded wards (n=75). The PSH on wards in SJUH and LGI achieved embedded status more quickly when compared to the other hospitals and within the 24 week period set for scaling up. The mean number of weeks for the three other hospitals (CAH, BGH and SGH) all exceeded this 24 week period. However, the overall mean for all wards (19.6) was within the anticipated 24 weeks.

| **Wards** | **Median (weeks)** | **Mean (weeks)** | **Min (weeks)** | **Max (weeks)** |
| --- | --- | --- | --- | --- |
| All  (n=75 wards) | 13 | 19.6 | 1 | 86 |
| SJUH  (n=31 wards) | 13 | 18 | 2 | 86 |
| CA  (n=3 wards) | 61 | 48 | 5 | 79 |
| LGI  (n=18 wards) | 9 | 18 | 1 | 57 |
| Barnsley  (n=11 wards) | 30.5 | 36 | 5 | 71 |
| Scarborough  (n=12 wards) | 18 | 25 | 6 | 85 |

**Number of weeks between start date and embedded date for embedded wards (n=75)**
