# Supplementary material for: Fidelity and the impact of patient safety huddles on teamwork and safety culture: an evaluation of the Huddle Up for Safer Healthcare (HUSH) project
Source: BMC Health Serv Res. 2021 Oct 1;21:1038. doi: 10.1186/s12913-021-07080-1 (PMC8487146; doi:10.1186/s12913-021-07080-1)
Supplement: Supplementary file 3 — Additional file 3. Sample of TSC survey sizes: pre and post embedded. [file 12913_2021_7080_MOESM3_ESM.docx]

**Appendix 3 Sample sizes for TSC survey: pre and post embedded**

| **Ward name** | **Pre-embedded** | **Post-embedded** |
| --- | --- | --- |
| 1 | 24 | 13 |
| 2 | 14 | 22 |
| 3 | 20 | 21 |
| 4 | 22 | 19 |
| 5 | 28 | 35 |
| 6 | 28 | 23 |
| 7 | 40 | 22 |
| 8 | 16 | 30 |
| 9 | 14 | 14 |
| 10 | 34 | 22 |
| 11 | 37 | 20 |
| 12 | 45 | 31 |
| 13 | 37 | 18 |
| 14 | 21 | 22 |
| 15 | 31 | 18 |
| 16 | 14 | 15 |
| 17 | 10 | 14 |
| 18 | 16 | 17 |
| 19 | 23 | 16 |
| 20 | 22 | 16 |
| 21 | 11 | 16 |
| 22 | 12 | 19 |
| 23 | 21 | 18 |
| 24 | 17 | 15 |
| 25 | 12 | 12 |
| 26 | 15 | 20 |
| 27 | 19 | 21 |
| 28 | 12 | 22 |
| 29 | 24 | 21 |
| 30 | 13 | 17 |
| 31 | 60 | 63 |
| 32 | 29 | 35 |
| 33 | 17 | 8 |
| 34 | 19 | 15 |
| 35 | 20 | 17 |
| 36 | 18 | 18 |
| 37 | 18 | 16 |
| 38 | 20 | 19 |
| 39 | 24 | 16 |
| 40 | 20 | 17 |
| 41 | 18 | 20 |
| 42 | 49 | 21 |
| 43 | 8 | 14 |
| 44 | 15 | 19 |
| 45 | 23 | 17 |
| 46 | 33 | 30 |
| 47 | 16 | 23 |
| 48 | 31 | 15 |
| 49 | 29 | 28 |
| 50 | 24 | 13 |
| 51 | 16 | 17 |
| 52 | 26 | 19 |
| 53 | 24 | 20 |
| 54 | 20 | 16 |
| 55 | 15 | 20 |
| 56 | 15 | 26 |
| 57 | 17 | 11 |
| 58 | 18 | 40 |
| 59 | 26 | 18 |
| 60 | 23 | 24 |
| 61 | 13 | 22 |
| 62 | 16 | 16 |
| 63 | 29 | 16 |
| 64 | 26 | 54 |
| 65 | 23 | 8 |
| 66 | 27 | 33 |

*Table 1: Sample sizes for TSC pre and post embedded*
