# Supplementary material for: Fidelity and the impact of patient safety huddles on teamwork and safety culture: an evaluation of the Huddle Up for Safer Healthcare (HUSH) project
Source: BMC Health Serv Res. 2021 Oct 1;21:1038. doi: 10.1186/s12913-021-07080-1 (PMC8487146; doi:10.1186/s12913-021-07080-1)
Supplement: Supplementary file 4 — Additional file 4. Teamwork and Safety Culture survey. [file 12913_2021_7080_MOESM4_ESM.docx]

###
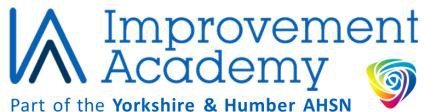
Date: ……..........................

**Teamwork and Safety Climate Survey**

Please answer the following items with respect to your specific unit or clinical area. Choose your responses using the scale below by placing “X” in the relevant box:

| **A** | **B** | **C** | **D** | **E** | **F** |
| --- | --- | --- | --- | --- | --- |
| **Disagree Strongly** | **Disagree Slightly** | **Neutral** | **Agree Slightly** | **Agree Strongly** | **Not Applicable** |

| **TEAMWORK CLIMATE** | | | | | | |
| --- | --- | --- | --- | --- | --- | --- |
| **1.** Nurse input is well received in this clinical area. | **A** | **B** | **C** | **D** | **E** | **F** |
| **2.** In this clinical area, it is difficult to speak up if I perceive a problem with patient care. | **A** | **B** | **C** | **D** | **E** | **F** |
| **3.** Decision-making in this clinical area utilises input from relevant personnel. | **A** | **B** | **C** | **D** | **E** | **F** |
| **4.** The doctors and nurses here work together as a well-coordinated team. | **A** | **B** | **C** | **D** | **E** | **F** |
| **5.** Disagreements in this clinical area are resolved appropriately (i.e. , not *who* is right, but *what* is best for the patient). | **A** | **B** | **C** | **D** | **E** | **F** |
| **6.** I am frequently unable to express disagreement with the medical staff here. | **A** | **B** | **C** | **D** | **E** | **F** |
| **7.** It is easy for personnel here to ask questions when there is something that they  do not understand. | **A** | **B** | **C** | **D** | **E** | **F** |
| **8.** I have the support I need from other personnel to care for patients. | **A** | **B** | **C** | **D** | **E** | **F** |
| **9.** I know the first and last names of all the personnel I worked with during my last  shift. | **A** | **B** | **C** | **D** | **E** | **F** |
| **10.** Important issues are well communicated at shift changes. | **A** | **B** | **C** | **D** | **E** | **F** |
| **11.** Briefing personnel before the start of a shift (i.e. to plan for possible  contingencies) is important for patient safety. | **A** | **B** | **C** | **D** | **E** | **F** |
| **12.** Briefings are common in this clinical area. | **A** | **B** | **C** | **D** | **E** | **F** |
| **13.** I am satisfied with the quality of collaboration that I experience with medical staff  in this clinical area. | **A** | **B** | **C** | **D** | **E** | **F** |
| **14.** I am satisfied with the quality of collaboration that I experience with nurses in this  clinical area. | **A** | **B** | **C** | **D** | **E** | **F** |

| **SAFETY CLIMATE** | | | | | | |
| --- | --- | --- | --- | --- | --- | --- |
| **15.** The levels of staffing in this clinical area are sufficient to handle the number of patients. | **A** | **B** | **C** | **D** | **E** | **F** |
| **16.** I would feel safe being treated here as a patient. | **A** | **B** | **C** | **D** | **E** | **F** |
| **17.** I am encouraged by my colleagues to report any patient safety concerns I may have. | **A** | **B** | **C** | **D** | **E** | **F** |
| Personnel frequently disregard rules or guidelines (e.g. hand washing, treatment  **18.** protocols/clinical pathways, sterile fluid, etc.) that are established for this clinical area. | **A** | **B** | **C** | **D** | **E** | **F** |
| **19.** The culture in this clinical area makes it easy to learn from the errors of others. | **A** | **B** | **C** | **D** | **E** | **F** |
| **20.** I receive appropriate feedback about my performance. | **A** | **B** | **C** | **D** | **E** | **F** |
| **21.** Medical errors are handled appropriately here. | **A** | **B** | **C** | **D** | **E** | **F** |

**PTO**🡺

| **A** | **B** | **C** | **D** | **E** | **F** |
| --- | --- | --- | --- | --- | --- |
| **Disagree Strongly** | **Disagree Slightly** | **Neutral** | **Agree Slightly** | **Agree Strongly** | **Not Applicable** |

| **22.** I know the proper channels to direct questions regarding patient safety in this clinical area. | **A** | **B** | **C** | **D** | **E** | **F** |
| --- | --- | --- | --- | --- | --- | --- |
| **23.** In this clinical area, it is difficult to discuss errors. | **A** | **B** | **C** | **D** | **E** | **F** |
| **24.** Hospital management does not knowingly compromise the safety of patients. | **A** | **B** | **C** | **D** | **E** | **F** |
| **25.** This organisation is doing more for patient safety now, than it did one year ago. | **A** | **B** | **C** | **D** | **E** | **F** |
| **26.** Leadership is driving us to be a safety-centred organisation. | **A** | **B** | **C** | **D** | **E** | **F** |
| **27.** My suggestions about safety would be acted upon if I expressed them to management. | **A** | **B** | **C** | **D** | **E** | **F** |

|  | **Excellent** | **Very Good** | **Acceptable** | **Poor** | **Failing** |
| --- | --- | --- | --- | --- | --- |
| **28**. Please give your unit an overall grade on patient safety. |  |  |  |  |  |

### Have you ever completed this survey before Yes No Don’t know

**BACKGROUND INFORMATION**

**Position (mark only ONE)**

| **Band 5 Staff Nurse** |  | **Health Care Assistant** |  | **Porter** |
| --- | --- | --- | --- | --- |
|  |  |  |  |  |
| **Band 6 Staff Nurse** |  | **Housekeeper** |  | **PT/OT/Speech** |
|  |  |  |  |  |
| **Band 7 Staff Nurse** |  | **Junior Doctor** |  | **Technician (e.g. EKG,Lab,Radiology)** |
|  |  |  |  |  |
| **Clinical Support Worker** |  | **Medical Administrator** |  | **Unit Asst. / Clerk / Secretary** |
|  |  |  |  |  |
| **Consultant** |  | **Medical Registrar** |  | **Ward Clerk** |
|  |  |  |  |  |
| **Dietician** |  | **Pharmacist** |  | **Ward Manager** |

**Domestic Other**…........................................................................

**Experience in Organisation:**

| Less than 6 |  | 6 to11 |  | 1 to 2 |  | 3 to 7 |  | 8 to |  | 13 to |  | 21 or more |
| --- | --- | --- | --- | --- | --- | --- | --- | --- | --- | --- | --- | --- |
| months |  | months |  | years |  | years |  | 12 years |  | 20 years |  | years |

| **Unit/Clinical Area (please write in your unit title/location:** | | |
| --- | --- | --- |
|  | **Ward Number:** |  |

***Thank you for completing this survey***

***– your time and participation are greatly appreciated***
